# Development of a web tool to increase research literacy in underserved populations through public library partnerships

**DOI:** 10.1371/journal.pone.0246098

**Published:** 2021-02-03

**Authors:** Melissa A. Simon, Catherine A. O’Brian, Laura Tom, Q. Eileen Wafford, Shenita Mack, Samuel R. Mendez, Magdalena Nava, Rabih Dahdouh, Rachelle Paul-Brutus, Kathryn H. Carpenter, Barbara Kern, Kristi L. Holmes

**Affiliations:** 1 Center for Health Equity Transformation and Department of Obstetrics & Gynecology, Feinberg School of Medicine, Northwestern University, Chicago, Illinois, United States of America; 2 Galter Health Sciences Library & Learning Center, Feinberg School of Medicine, Northwestern University, Chicago, Illinois, United States of America; 3 Chicago Public Library, Chicago, Illinois, United States of America; 4 Chicago Department of Public Health, Chicago, Illinois, United States of America; 5 University Library, University of Illinois-Chicago, Chicago, Illinois, United States of America; 6 The John Crerar Library, University of Chicago, Chicago, Illinois, United States of America; 7 Department of Preventive Medicine, Feinberg School of Medicine, Northwestern University, Chicago, Illinois, United States of America; Columbia University, UNITED STATES

## Abstract

**Objective:**

Inadequate diversity in clinical trials is widely recognized as a significant contributing factor to health disparities experienced by racial/ethnic minorities and other diverse populations in the US. To address this in a scalable way, we sought to develop a web tool that could help enhance underserved minority participation in clinical research.

**Methods:**

We used our research literacy support flashcard tool as the initial prototype for human-centered design and usability testing of the web tool Health for All in public library settings. After forming partnerships with leadership from Chicago Public Libraries (CPL), local medical libraries, and the Chicago Department of Public Health, we conducted seven iterative design sessions with focus groups of library patrons and library staff from six CPL branches serving underserved communities followed by two rounds of usability testing and website modification.

**Results:**

Based on the qualitative research findings from Design Sessions 1–7, we enacted the design decision of a website that was a hybrid of fact-filled and vignette (personal stories) paper prototypes divided into 4 modules (trust, diversity, healthy volunteers, pros/cons), each with their own outcome metrics. The website was thus constructed, and navigation issues identified in two rounds of usability testing by library patrons were addressed through further website modification, followed by the launch of a beta version of a hybridized single-scrolling and guided module prototype to allow further development with website analytics.

**Conclusions:**

We report the development of Health for All, a website designed to enhance racial/ethnic minority participation in clinical trials by imparting research literacy, mitigating distrust engendered by longstanding racism and discrimination, and providing connections to clinical trials recruiting participants.

## Introduction

Precision medicine is "an emerging approach for disease treatment and prevention that takes into account individual variability in genes, environment, and lifestyle for each person" [1]. The goal of the practice of precision medicine in the US is to give health care providers the information they need to make customized recommendations for their patients [2]. A significant barrier to the realization of this goal is the underrepresentation of racial/ethnic minorities in clinical research in the US [3–5]. This lack of parity is the impetus for national initiatives such as the All of Us Program, which aims to increase diversity in clinical trials and biobanking initiatives in order to support more equitable representation in clinical research and bring the benefits of advances in healthcare forged by clinical research equitably to all, including currently underrepresented minority populations [2].

There are myriad barriers to participation in clinical research experienced by underrepresented minorities, such as cost (e.g., lack of health insurance, high out-of-pocket expenses), exclusion criteria (e.g., comorbid conditions, functional status and age), and logistics (e.g., transportation and time commitment or scheduling challenges) [6,7]. A major barrier is the distrust engendered in underrepresented minorities by longstanding experiences of racism and discrimination. This includes the heinous deception and mistreatment of African Americans in the Tuskegee syphilis study and other exploitative studies in the historical record [8]. Another significant barrier is limited research literacy, which includes lack of awareness of opportunities to participate in clinical research and what participation entails, and misperceptions about clinical research [5–7]. Further, limited research literacy and unfamiliarity with clinical research concepts presents the risk that individuals may make a decision about participating in research without adequately understanding the information presented in the informed consent process [5]. Advancing research literacy, particularly among underrepresented minorities, may avert or mitigate this risk, and at the same time enhance the diversity of participants in clinical research, e.g., by improving trust and empowering individuals to participate in research.

When developing health interventions in community settings, community-based approaches to human-centered design processes have been shown to align interventions such as web tools with the needs and contexts of the community members served and other local stakeholders [9,10]. We sought Chicago Public Library (CPL) partnership for Health for All development because public libraries are widely recognized as valuable and logical settings for community outreach, engagement and education, including in the domain of public health [11–13]. We now report on the development of Health for All, a web-based tool adapted from a Research Literacy Support (RLS) tool to the setting of public library branches via community-based, human-centered design and usability testing. Health for All is designed to both enhance research literacy among diverse library patrons and connect them with clinical trials.

## Materials and methods

### Research literacy support tool

In partnership with the Cancer Disparities Research Network (CDRN) and under the auspices of the NCI-funded Geographic and Biobanking Management Program (G/BMaP), we previously employed a user-centered design approach to develop a flashcard-based Research Literacy Support (RLS) communication tool for use by clinical trial recruiters to advance research health literacy among potential clinical research participants by serving as an interactive guide that enhances communication with the recruiters [5,14]. Development of the RLS tool is published elsewhere [5,14] but briefly, the development process involved three phases. Recognizing the essentiality of investigating the specific needs of intended users at the outset of a user-centered design process [15], a needs assessment and initial prototype creation was accomplished in Phase 1. Upon completion of Phase 1, the RLS tool consisted of a series of 34 cards organized into sections on basic research information, study-specific information, and research participants’ rights in addition to cards that addressed myths and truths about clinical research [14].

Phase 2 of RLS tool development entailed cognitive interviewing [5,16]. Phase 2 was comprised of two iterations of semi-structured interviews with potential research participants representing underrepresented racial/ethnic groups and experienced clinical research recruiters followed by modifications to the tool content and design [5].

In Phase 3 of RLS tool development, a usability survey was administered to potential research participants from underrepresented racial/ethnic groups after they reviewed the RLS flashcard prototype. The usability survey results indicated that the cards were informative, easy to read, and explained research concepts in simple language [5]. The RLS tool was thus found to be useful to both participants and researchers at initiating, guiding, and fostering communication between researchers and participants that may foster minority participation in clinical research [5]. This RLS flashcard-based tool formed the basis of the Health for All web tool to be adapted for public library settings in which to scale and reach a larger audience.

### Design thinking model

Our approach to the community-based, human-centered design and usability testing of the web tool Health for All from the flashcard RLS tool was guided by the Stanford d.school model of design thinking. This design thinking framework is termed human-centered design because it centers on empathy [17]. Sometimes referred to as user-centered design, human-centered design integrates a set of practices to understand users in order to build deep empathy with them, generate multiple ideas to help solve their problems, and arrive at a solution via rapid prototyping [18]. The Stanford model of design thinking entails iterative implementation of five stages: empathize, define, ideate, prototype, and test [19]. In this design thinking model, the five stages do not have to follow any specific order and they can often occur in parallel and be repeated iteratively [19]. In our iterative design process, *empathize* meant understanding the needs and experiences of the end users of the Health for All intervention, who are public library patrons, as well as those of public library staff/managers and our Chicago Public Library executive partners; *define* entailed understanding the diverse experiences and perspectives of public library patrons and library staff/managers about the intervention to allow human centered refinement of the tool; *ideate* involved nonjudgmentally generating multiple options to solve problems identified in the *define* stage; *prototype* entailed developing concrete representations of solutions identified in the *ideate* stage in the form of paper and web interface prototypes of the intervention; and *test* involved evaluating and garnering feedback on intervention prototypes.

### Partnership building for community-based development of Health for All

The Health for All tool was developed by the Northwestern University Center for Health Equity Transformation and the Northwestern University Galter Health Sciences Library and Learning Center, in partnership with Chicago Public Library (CPL), the University of Chicago John Crerar Library, the University of Illinois-Chicago Library of the Health Sciences-Chicago, and the Chicago Department of Public Health. Prior to meeting with CPL, we conducted background research on CPL infrastructure, visit statistics, and events, and we created user stories to conceptualize the context in which the tool would be used. We thus homed in on concepts/questions for initial project meetings with CPL related to technical resources, current scope of responsibilities for librarians, and current interactions with other university researchers. In the inaugural meeting, which was attended by representatives of our research team, CPL administration, the Chicago Department of Public Health, and leadership from local medical libraries, discussions centered on the goals and human-centered design approach of the project. Following the meeting, CPL would identify six diverse library branches in which to deploy the design-thinking process to develop Health for All, which would include focus group design sessions, usability testing, and deployment of iterative prototypes. The 6 library branches identified by CPL represented its northern, central and southern districts. Subsequent meetings were held between the research team and branch managers that involved project overviews and descriptions of the human-centered design process, followed by discussions about the needs of the librarians within the partnership.

### Environmental scan

Data pertaining to the participating library branches was obtained from CPL and included information about the types of books borrowed from each branch and statistics describing public accessing of various sections of the website of each branch. Observational visits were made to each of the library branches by the research team to note setting characteristics such as noise level and how the library patrons used the space, e.g., browsing books or newspapers, using computers, gathering and conversing with each other, etc. Important to web design methodology, all six branches shared the same computer technology, which was Windows desktop computers with Internet Explorer 11 as the only available web browser.

### Paper prototyping of the web interface

Paper prototyping is recognized as important to the efficiency of human-centered web design processes, both in terms of speed and cost containment. Paper prototypes are easily modifiable and thus can be used early in the design process to conduct rapid iterative testing of the terminology, navigation, content, page layout, and functionality of the interface and thereby identify and resolve problems prior to investments in programming [20–22]. To design the web tool to be employed in usability testing, we gathered input from focus groups and CPL partners, e.g., some of the resources deployed were recommended by CPL partners, and the structure of guiding questions was informed by focus group input. We conducted seven iterative design sessions with focus groups of library patrons and library staff at the six participating local public library branches (CPL Branches A-F); six design sessions were conducted sequentially at each branch in the order of A to F, and the seventh design session was held at CPL Branch A. A convenience sample of 3–6 patrons and library staff from each branch were recruited through flyers posted at bulletin boards within that branch and on-site by research assistants in the hour prior to the focus group; all persons 18 years of age and older were eligible to participate. Focus group participants recruited among library staff included the branch manager(s). In each session, the agenda included three items. The first item was the storyboarding of a typical library visit; the second item was a facilitated discussion to glean focus group participants’ awareness and opinions regarding clinical trial participation and the underrepresentation of racial/ethnic minorities and other groups in clinical trials; and the third item was eliciting focus group responses to the RLS flashcard tool presented as a paper prototype of a web interface. All sessions were conducted in English, were audio recorded, and lasted approximately one hour. At each session, one research team member served as the moderator, and another took observational notes about participant movement, tone, and gestures throughout the session. Based on the audio recording and observational notes, summaries of each session were written and used to inform iterative refinement of the paper prototype. Data were analyzed by employing qualitative methods as previously described [23] to identify codes and generate prevalent themes. Focus groups sessions were held iteratively until thematic saturation–where no new themes emerged.

### Web tool usability testing

The methodology used in the usability testing phase of the study was think-aloud [24] combined with audio recording, screen capture, and observation, as described in detail below. The general need for users to navigate with sufficient ease to find information in an acceptable timeframe guided our web tool usability testing approach, as did recognition that our target audience includes persons of low literacy and limited computer skills, underscoring the importance of simplified navigation strategies to optimize accessibility [25]. Together with a technical consultant, we built a single-page web tool with a long-scrolling layout in Wordpress (Version 1) and an InVision App click-through wireframe of a multi-page prototype with four guided modules, (e.g., trust in clinical trials), for the initial round of usability testing on a web interface at CPL Library Branches A and D. We recruited a convenience sample of usability testing participants by posting flyers at these library branches and by on-site recruitment by research assistants the day of the testing session; all persons 18 years of age and older were eligible to participate. The usability testing script asked participants to give their first impressions on certain sections of the single-page WordPress build, find specific information, give an overall impression of the website, and then make a comparison to the multi-page prototype. We ran these tests with one facilitator asking questions. We used a voice recorder to document the session. We used Panopto screen capture to document on-screen cursor movement. There were two note takers present to document the participants’ body language and tone of voice, any assistance required to complete the assigned usability tasks, and overall routes through the website. The records of the note takers revealed those instances where responses to the facilitator did not align with observed user experiences, e.g., where a user described website navigation as easy but required assistance with this. Thus, we assessed ease of use by both qualitative interviewing and observation. Based on coding and thematic analysis of notes taken and participant responses [23], we made adjustments to the single-page web tool prototype iteratively during the first round of usability testing to improve user experiences, resulting in Version 2 of the web tool. We conducted the second round of usability testing similarly at CPL Library Branches D and G using a multi-page prototype that incorporated the improvements made to the single-page prototype during the first round of usability testing, with iterative improvements made to the WordPress build based on documented user experiences, resulting in Version 3.

### Human subjects research

All research reported here involving human participants, i.e., library patrons and staff, was approved by the Institutional Review Board (IRB) of Northwestern University and was conducted according to the principles expressed in the Declaration of Helsinki. All participants provided written informed consent prior to study participation.

## Results

### Characteristics of the participating CPL library branches and the neighborhoods they serve

The key library characteristics of the six CPL library branches selected by CPL for participation in the human-centered design of Health for All are shown in **Table 1** (Branches A-F), which reflects 2017 data. Activity levels varied widely across the six branches, as evident from comparing the size of circulation (i.e., lending activity), which ranged from 15,017 to 129,314, and the number of visitors (range, 33,578–157,976). In addition to these environmental differences, germane to the planned web tool development in the public library setting was computer accessibility. Table 1 shows that the six branches all had >10,000 computer sessions in 2017, indicating the appropriateness of these branches as web tool development settings.

**Table 1:** Library characteristics at CPL branches serving as settings for human-centered design and usability testing of Health for All^1^.

Branch	Circulation	Computer Sessions	Visitors	Holds Filled	Holds Placed
A	23,192	24,112	33,578	930	8,362
B	121,534	30,645	157,976	1,567	44,415
C	25,300	10,157	54,963	1,130	10,147
D	129,314	24,105	144,228	2,319	59,244
E	56,112	17,537	71,204	1,024	13,851
F	15,017	16,810	56,626	205	6,097
G	634,570	287,129	1,233,309	21,051	836,615

^1^Data correspond to 2017 and were provided by CPL.

**Table 2** shows the demographic characteristics of the neighborhoods served by the selected CPL branches (CPL Branches A-F). All six branches served neighborhoods where the majority of residents were racial/ethnic minorities, as reflected in the demographic characteristics of the neighborhoods in Table 2. The racial/ethnic and socioeconomic diversity across the six neighborhoods can also be gleaned from Table 2. Two branches (A, F) representing the Southern and Central districts of Chicago were in neighborhoods that were >90% Black or African American, one branch (E) was in a neighborhood that was >80% Hispanic or Latino, and three branches (B-D) were in neighborhoods which were 45–50% Hispanic or Latino, 29–45% White, and diverse across socioeconomic characteristics, particularly the percentage of residents who were college graduates. Thus, the library characteristics of the selected CPL branches and the racial/ethnic and socioeconomic diversity across the communities they serve both support the appropriateness of these six branches as settings for community-based, human centered design of a web tool intended to serve health disparities populations, and observational visits to the library branches by research team members were confirmatory of this.

**Table 2:** Demographic characteristics of Chicago neighborhood locations of CPL branches participating in human-centered design and usability testing of Health for All^1^.

Branch (District)	% Black or African American	% Hispanic or Latino	% White	% Other	% Not Graduating from High School	% College Graduate	Median Income	% Un-employed
A (Southern)	96	1	1	1	15	15	31K	24
B (Northern)	4	48	29	18	75	30	52K	9
C (Southern)	5	50	45	0	15	17	44K	6
D (Northern)	5	45	45	5	13	50	73K	5
E (Central)	1	85	6	8	44	9	39K	11
F (Central)	93	2	3	2	15	27	30K	21
G (Central)^2^	11	6	62	21	2	78	131K	5

^1^The data source for this table is the Chicago Health Atlas (https://www.chicagohealthatlas.org/community-areas) of the Chicago Department of Public Health.

^2^Branch G is located in a central business district.

### Human-centered design of the web tool Health for All

**Fig 1:**
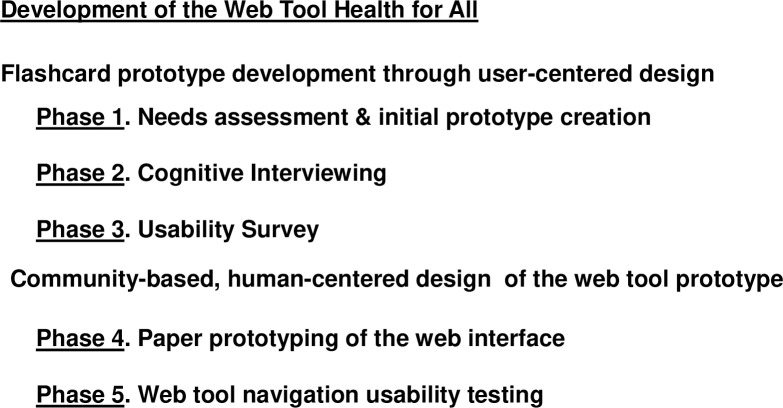
Development of the web tool Health for All.

**Fig 2:**
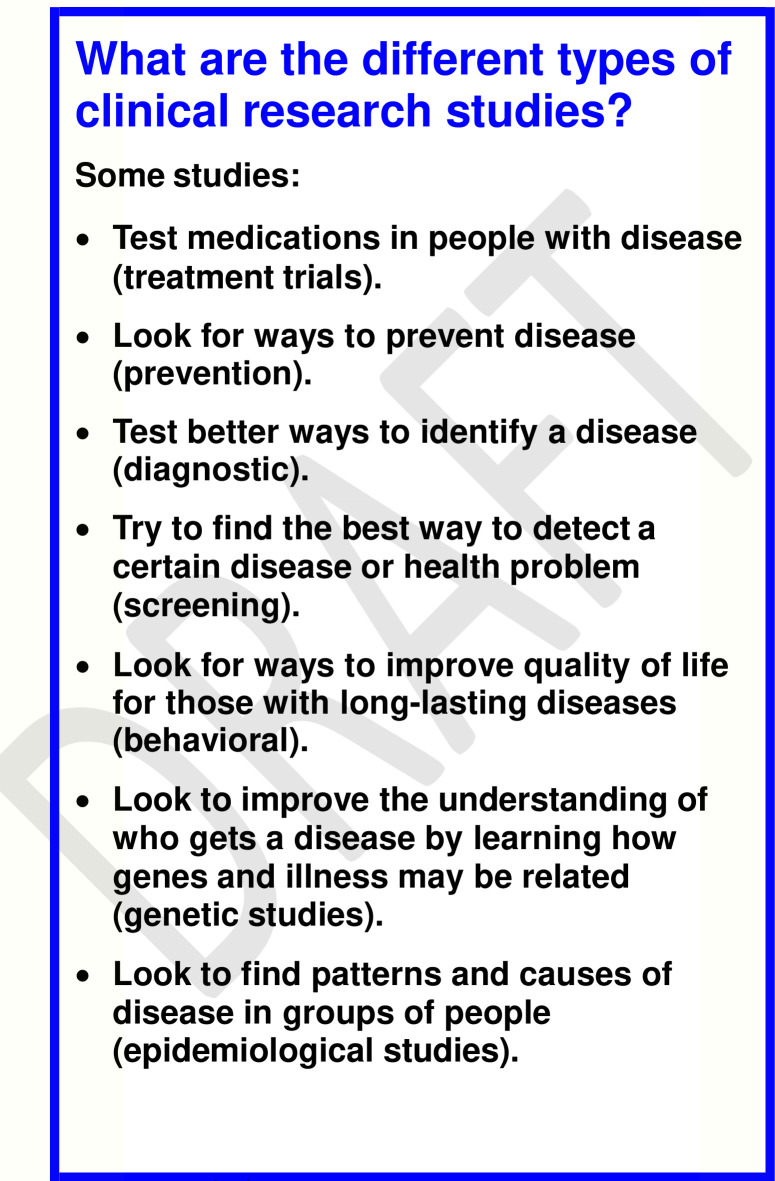
Flashcard example from the Research Literacy Support (RLS) tool.

Phase 4 of development of the web tool Health for All (**Fig 1**) entailed seven iterative community-based, human-centered design sessions with paper prototyping of the web interface at CPL Branches A-F. **Table 3** shows the demographic characteristics of the focus groups participating in iterative human-centered design sessions at the CPL branches. We note that the total number of participants was not affected by cancellation or no-shows in any of the focus groups, because focus group recruitment was primarily conducted on-site shortly before each session began. The initial paper prototype displayed the content of the Research Literacy Support (RLS) flashcard tool [5]; for an example of a flashcard in the tool, see **Fig 2**.

**Table 3:** Demographic characteristics of focus groups participating in human-centered design of the web tool Health for All.

Design Session (Branch)	Total Number of Participants	Black or African American	Hispanic or Latino	Asian	Non-Hispanic White	Men	Women	Age (yrs)
1 (A)	5	5	0	0	0	1	4	30–59
2 (B)	4	0	0	2	2	0	4	40–70+
3 (C)	5	0	3	1	1	2	3	18–69
4 (D)	6	1	0	1	4	2	4	30–59
5 (E)	4	0	2	0	2	2	2	40–69
6 (F)	6	5	0	0	1	4	2	18–70+
7 (A)	3	3	0	0	0	1	2	30–69

#### Design Session 1

There was extensive discussion among focus group participants about barriers to participating in clinical trials faced by African Americans and other minorities and past experiences. Among the focus group participants, both library patrons and library staff expressed that they distrusted clinical research including clinical trials and referenced Henrietta Lacks and Tuskegee. Focus group participants viewed the paper prototype of Health for All as too formal, too impersonal, and too distant. The prototype elicited responses of distrust and skepticism and was considered uninviting. This general response was compounded by the distrust participants expressed about clinical trials and the medical community. Modifications of the tool recommended in the group discussion were to add contact information, incorporate personal stories, and make the tool modular for the users for customizable user experiences. Participants viewed the library setting favorably; they related that the social aspects of the library were a major draw, and that the availability of computers was important to them.

#### Design Session 2

**Fig 3:**
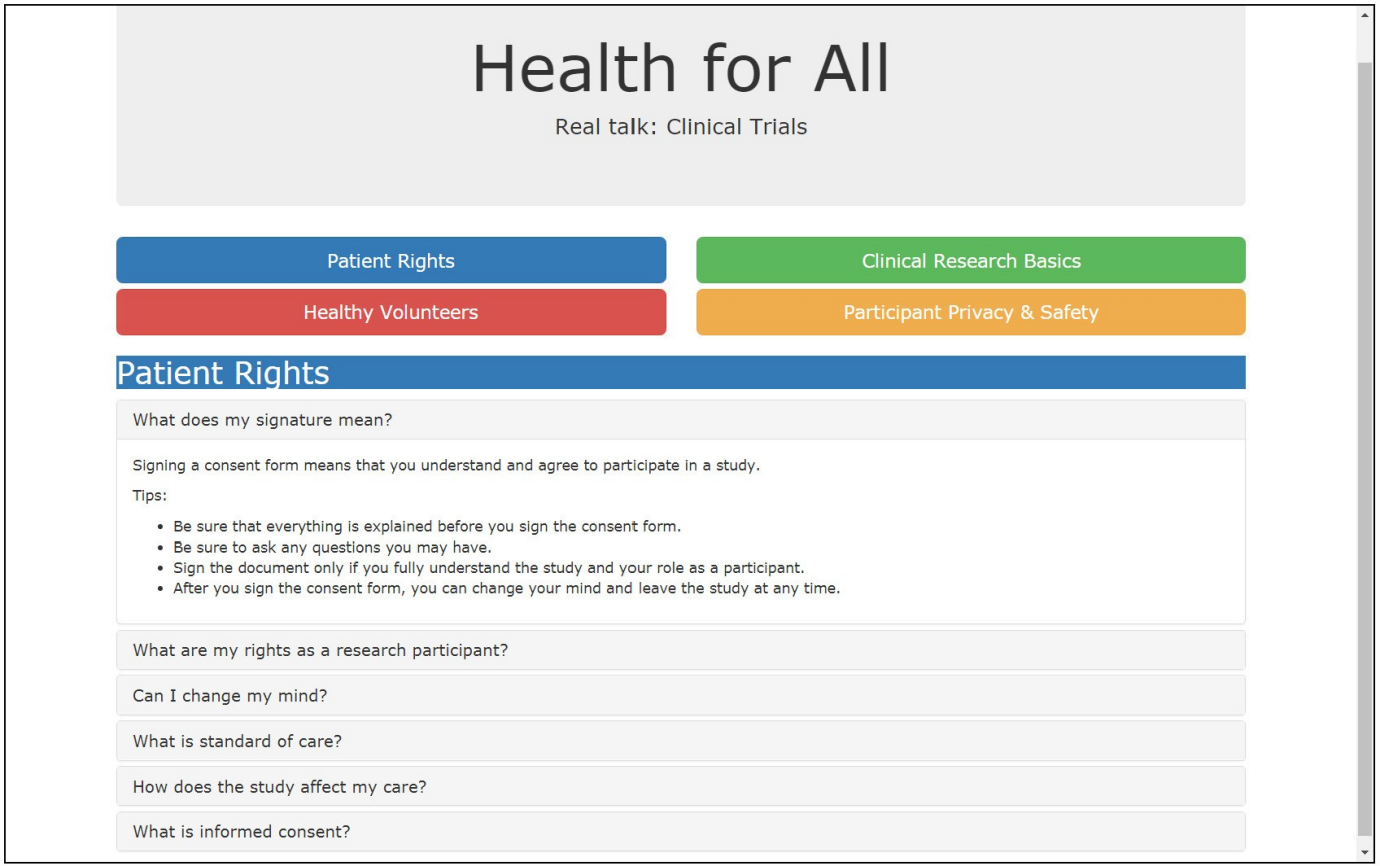
Fact-filled version of the Health for All prototype developed after design Session 2.

**Fig 4:**
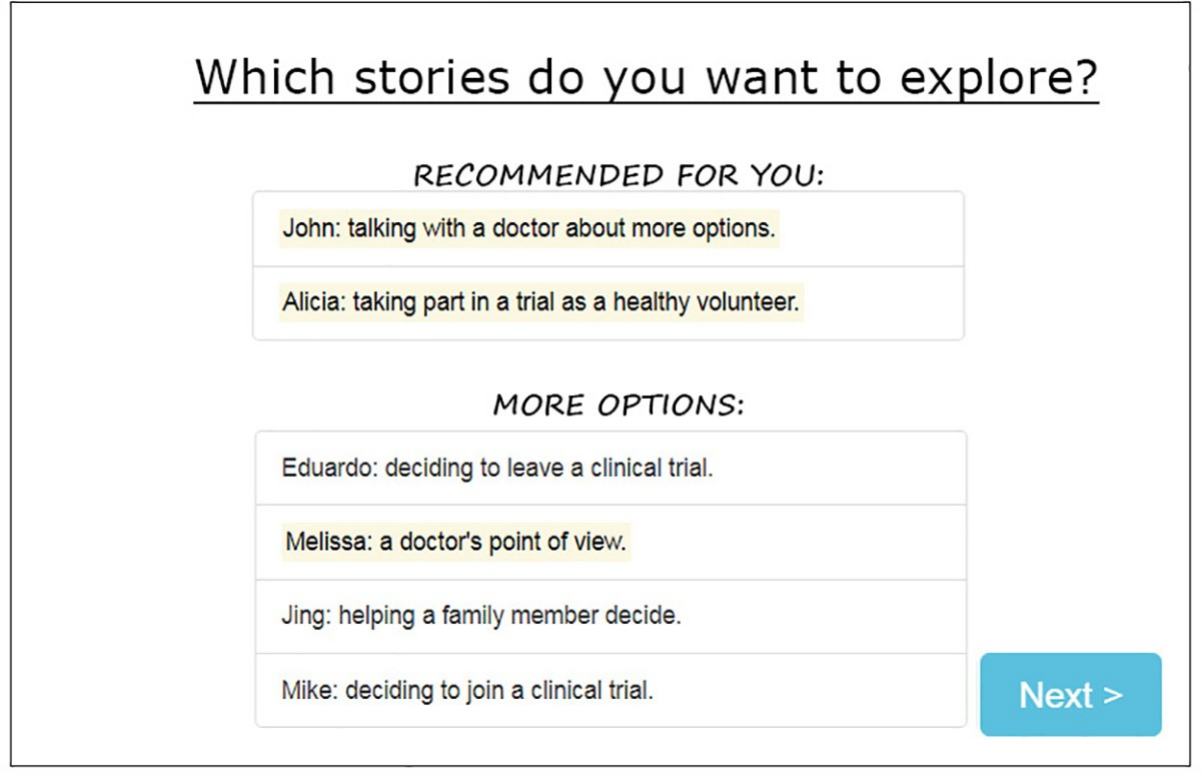
Vignette version of the Health for All prototype developed after design Session 2.

The strong distrust of clinical research and trials expressed in Design Session 1 was not expressed by this group. Multiple participants related personal or family experience with clinical trials, and they discussed having seen clinical trial recruitment notices on buses, in mailings from local universities, and on the community board of the library. Provider recommendation of clinical trials was described as the most important consideration in making a decision about whether or not to join clinical trials. Consonant with the level of trust in the medical community expressed by this group, participants described the paper prototype as "informative," "easy to understand," and "just fine." When asked, they stated that they did not find the tool to be “impersonal” or too “formal,” and they noted that they did not want a website that was overly simplistic. Similar to the tool feedback in Design Session 1, participants brought up that people have different learning styles, and they recommended having different options to meet these diverse preferences. In response to this feedback, we refined the prototype as a fact-filled version (**Fig 3**) and also created a vignette version of the prototype, which featured live-action video-based stories (**Fig 4**). Regarding the setting, participants shared that they generally come to the library for books and events, and less often for computer access.

#### Design Session 3

A notable theme in this session was the group’s perception of clinical trials as being pertinent only when a person is ill and a lack of awareness of participation of healthy volunteers in clinical trials. This was consistent with views expressed in Design Sessions 1 and 2, and indicated the importance of addressing this misperception in the content of Health for All. Participants did not distrust clinical trials and recognized diversity in clinical trials as important. Participants were presented fact-filled and vignette versions of the prototype, and both were deemed usable by the group, although the fact-filled version was viewed as better organized. There was much discussion about the target audiences for these prototype versions. The fact-filled version was viewed as more useful for someone seeking information quickly, whereas the vignette version was seen as more suitable for those who hold skepticism or distrust of clinical trials. The group emphasized the need for multiple formats to accommodate personal learning style, range of questions/concerns, education level, and accessibility needs. This raised the question of how to filter audiences to a more personalized experience. With respect to the library setting, multiple participants noted that this library branch was quieter than most and a good venue for study and computer access. Given the very different perspectives of the focus groups participating in Design Sessions 1–3, we decided not to iteratively modify the prototypes based on focus group feedback for the remaining sessions and instead make further modifications at the end of this phase of web tool development.

#### Design Session 4

Fear and distrust around clinical trials were discussed, but not necessarily distrust for research or the medical system. The fear and distrust were centered on the risk of taking a medication where the full effects and long-term risks are unknown. The vignette version of the prototype seemed preferred by the group overall. Notably it was viewed as being more persuasive and convincing than the fact-filled version. Further, participants noted that they would want to speak with an informed person before engaging the fact-filled version. However, participants still thought that the information in the fact-based prototype could have more answers to the kinds of questions people would have in an actual clinical trial. Focus group members used the library for diverse purposes, such as access to children’s programming and a quiet place to study; many discussed using the library to look into medication, medication interactions, and new diagnoses.

#### Design Session 5

The participants of this focus group expressed that they were very receptive to clinical trials. However, multiple participants noted the need to be within the included age range and to have the studied ailment as limiting enrollment opportunities. They also expressed interest in new clinical trials as they get older and have new diagnoses, e.g., hearing loss. The group was not aware that racial/ethnic minorities are underrepresented in clinical trials. Participants liked both versions of the prototype, and there was not a consensus about which was preferred. One participant preferred the fact-filled version because they enjoy reading whereas another compared it to a user agreement that people never read. The group agreed that the vignette version helped with added context, but one participant said it reminded them of work trainings. Participants in this design session all reported using the library for social/recreational reasons, and their visit frequency ranged from twice a month to every day.

#### Design Session 6

A theme of distrust of clinical trials arose in this session. The group consensus was that the African American community, particularly men, don't trust clinical trials out of historical and political concerns. There was also a consensus that people of color are largely excluded from clinical trials, because they are not recruited, and the trials don't provide their communities access. The group felt that the tool would be useful for those who read but would not reach those with other learning styles or short attention spans. Alternative formats suggested included audio (podcasts) and video clips of conversations. A live format was also recommended to sustain interest. Regarding the library setting, participants said that they regularly visited the library to browse/borrow DVDs, books, newspapers, and to access computers.

#### Design Session 7

Participants were aware of the need for more diverse clinical trials. Multiple participants pointed out that the underrepresentation of minorities in clinical trials results from multiple barriers in addition to lack of knowledge, e.g., socioeconomic factors, language barriers, and lack of diverse providers were mentioned. Participants felt strongly that personal vignettes and government resources were preferred over other content as trustworthy sources. Due to time constraints, library utilization was not discussed.

New themes did not arise after the sixth design session, suggesting that saturation was reached and supports that the sizes of the individual focus groups and the number of design sessions held were sufficient for completion of this phase of development after seven sessions. The data from Design Sessions 1–7 led to our team’s design decision of a Health for All website that was a hybrid of the fact-filled and vignette paper prototypes divided into 4 modules (trust, diversity, healthy volunteers, pros/cons), each with their own outcome metrics. The Trust module served to address the theme of distrust of clinical trials that was emphasized at some design sessions. The Diversity module was to address the need for increased awareness of the underrepresentation of racial/ethnic minorities in clinical trials. The Healthy Volunteers module addressed the fact that our design sessions showed us that potential clinical trial healthy volunteers would have different informational needs and different concerns than other users. Finally, the Pros/Cons module was chosen because design session participants commonly described someone looking at this website after a conversation with a doctor, or to prepare for a conversation with a doctor about a clinical trial. Next, our technical consultant completed an InVision App click-through wireframe of a “Guided Modules” prototype and a WordPress build of a “Single Scrolling Page Modules” prototype. The latter was a fully functional website, except that it did not include the feedback questions we developed for the end of each module. Initial feedback on these prototypes was provided by leadership from local medical libraries and CPL, CPL library branch managers, and other members of the partnership. There was a positive consensus on the readability and understandability of the text and ease of navigation; minor modifications were made to wording and icon choice in the prototypes as a result of this feedback.

### Web tool usability testing

Phase 5 of the development of the web tool Health for All was the usability testing of the website prototype by CPL library patrons (Fig 1); this entailed two rounds of testing and website modification. Usability testing was conducted as one-on-one sessions between each participant and a member of the research team; in addition, another member of the research team took notes at these sessions. The demographic characteristics of the convenience sample of library patrons participating in the first round of usability testing are shown in **Table 4**. Based on observations that five participants are required per formative test cycle to detect over 80% of issues in usability testing [26], we conducted two iterative cycles of usability testing with totaling 13 (Round 1) and 11 (Round 2) participants.

**Table 4:** Demographic characteristics of library patrons participating in usability testing of the web tool Health for All.

Usability Round (Branch)	Total Number of Participating Library Patrons	Black or African American	Hispanic or Latino	Asian	Non-Hispanic White	Men	Women	Age (range, in yrs)
1 (A)	3	2	0	0	1	3	0	18–59
1 (D)	10	3	3	2	2	6	4	18–69
2 (D)	7	1	3	1	2	6	1	40–70+
2 (G)	4	3	1	0	0	3	1	18–59

The usability testing script is provided in **Table 5**. For the list of tasks performed, see **Table 6**.

**Table 5:** Usability testing script for Health for All development.

Thank you so much for joining us today. I'll start things of by giving you a little info about what we'll be doing, and give you time to ask any questions before we get started.
Today we're asking you to give us feedback on a draft of a website. Our goal is to see how easy or hard you find the website to use. I'll ask questions and give you instructions about what to do on the website. My teammate here will help take notes. We'll also be recording what happens on screen and voice recording this session.
During this session, we want you to think out loud as you go through the website. There are no right or wrong answers, and I won't really offer any hints or help to go through the website. I might ask you to voice your thoughts every so often.
If you have any questions while you're working, please let me know. I might ask you for more information, put you on the right track, or just move us on to the next task.
Your name will not be reported with any of the data from this test. And just a reminder that you can leave or stop this feedback session at any time. Do you have any questions before we begin?
Question: On first glance, what information do you expect to find on this web site?
Tasks 1―10
Question: Overall, how easy or hard would you say it was to understand the information on this website?
Question: Overall, how easy or hard was it trying to find specific information on this website?
Question: How much did you trust the information you saw on this website? Why?
Question: If you had questions after seeing this website, where do you think you could go for the answers?
Question: We are going to show you another version of this website, and we want to know if you'd prefer to use that version or the version you just saw.

**Table 6:** List of tasks in the usability testing phase of Health for All development.

Task 1: On the home page, find the link that will take you to section of this website that talks about healthy participants in clinical trials. Do not click on the link to that page yet.
Task 2: Go to the page that explains why healthy volunteers are important.
Task 3: Go to the page that explains… • Why are healthy volunteers important • Why should I volunteer if I’m healthy • Why would I not want to participate • How do healthy volunteers find the right study • That has links to other websites. Feel free to click on any links or buttons if you need to.
Task 4: Find a link to a book from Chicago Public Library and visit that website.
Task 5: Navigate back to the homepage of the Health for All website.
Task 6: Find a link to Northwestern's clinical trials listing, and visit that page.
Task 7: Navigate back to the home page of the health for all website.
Task 8: From the home page, find a new way to navigate to the section of the website that talks about healthy participants in clinical trials.
Task 9: If you wanted to find out what a "control group" means in a clinical trial, where would you go on this website? Try to find the definition of that term, "control group", in this site.
Task 10: Go back to the home page and browse around any other sections of the website that interest you for a few minutes.

**Fig 5:**
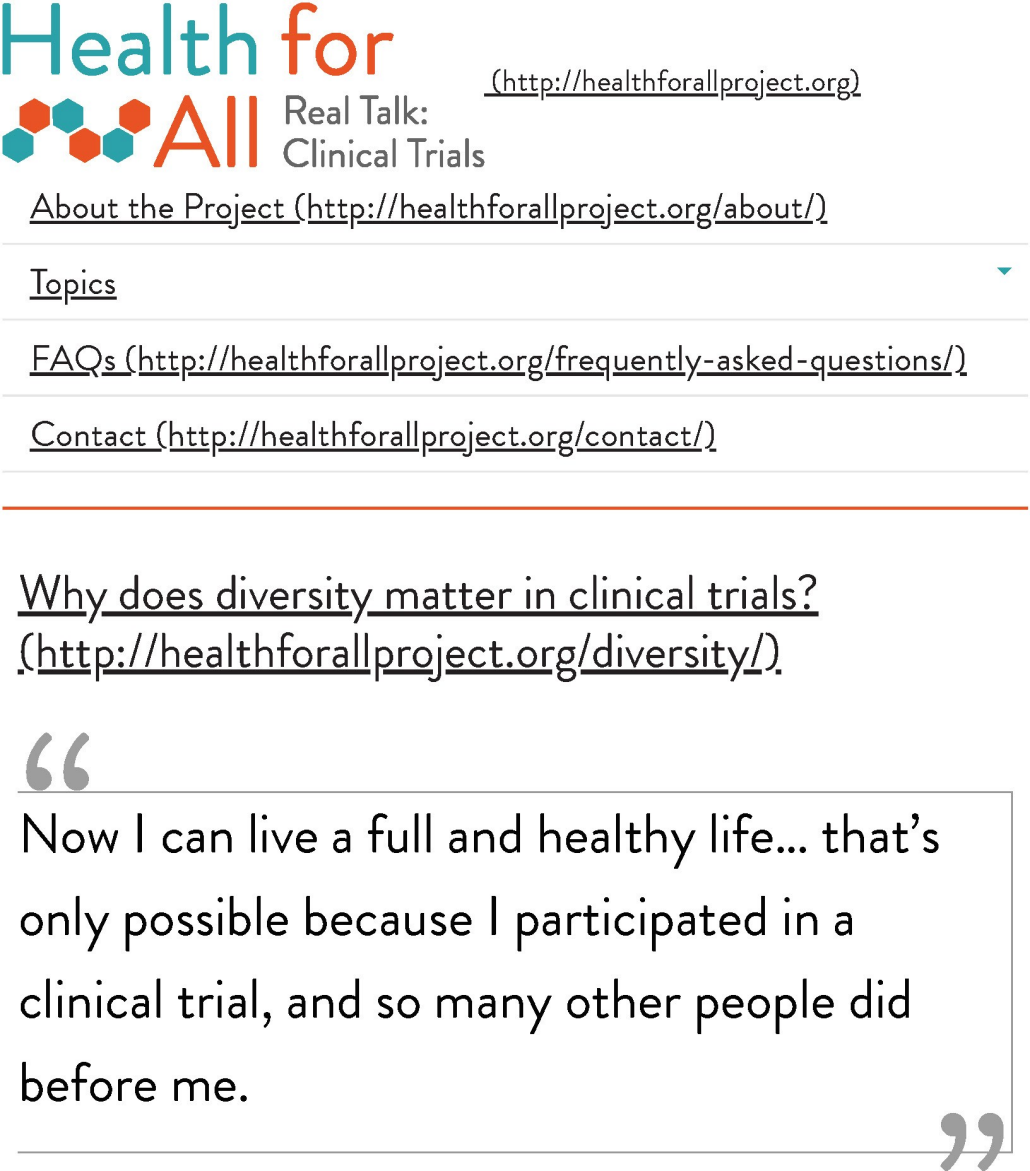
Version 2 of the guided module Health for All prototype.

In Round 1 of usability testing, participants were each given ten tasks that required navigation of the single scrolling website to specific content or feedback about understanding of specific content, and then were asked to compare the website with the guided module prototype. When asked, most (12/13) indicated that they found the content in the website easy to understand; it was noted that the content was free of medical jargon and accessible to native English speakers. When asked specifically about the definition of “control group” on the website, 11/13 found it easy to understand. The website was considered a trustworthy source of information by 8/13 participants; two participants noted that they would need contact information to trust the website, and one noted that they would trust it only if it was recommended by their physician. The website was viewed as difficult to navigate by 6/13 participants and easy to navigate by the rest. The key navigation-related usability issues revealed in the first round of testing included: 1) the lack of a home button made navigation to the home page difficult for some, as navigation entailed clicking on the Health for All logo; 2) hyperlinks were not readily visualized by some because they were not sufficiently distinct; 3) font size was too small for some to easily decipher; and 4) there was not a one-click path to view all external resources. The majority (8/13) preferred the guided modules platform, finding it more compact, better organized and easier to quickly navigate; those who preferred the single scrolling platform indicated they found it simpler to navigate. Issues identified in this round of usability testing were remedied by further refinement of the website and guided modules prototypes, resulting in version 2 of these platforms (**Fig 5**).

Eleven library patrons participated in Round 2 of usability testing with version 2 of the single scrolling website and the guided module prototype; their demographic characteristics are shown in Table 4. Participants were asked to complete the same ten tasks with the single scrolling website as those employed in Round 1 and to compare the single scrolling website with the guided module prototype. The majority (8/11) found the website content easy to understand, 8/11 considered the definition of “control group” easy to understand, 6/11 viewed the website as a trustworthy source of information, and 6/11 found the website easy to navigate. The guided module prototype was the platform preferred by 4/11 participants, while 6 participants did not express a preference. Minor adjustments were made to version 2 following usability testing, resulting in version 3, which we launched as a beta version of a hybridized single-scrolling and guided module prototype to allow further development with website analytics. The Health for All web tool is available for public access at https://healthforallproject.org/.

## Discussion

In this report, we describe the iterative development of the web tool Health for All through community-based participatory research and human-centered design in public library settings. The inclusion of Black or African American, Hispanic or Latino, and other diverse library patrons as participants in the human-centered design and usability testing phases of tool development was an intentional means of achieving accessibility and engagement among diverse racial/ethnic groups, as the purpose of the tool is to increase diversity in clinical trials by enhancing research literacy among diverse library patrons and simultaneously giving them access to clinical trial enrollment opportunities. Human-centered design of the web tool entailed seven iterative focus group sessions held at six public library branches serving diverse neighborhoods in Chicago. At these sessions, participants discussed how they use the library and how they view clinical research and the underrepresentation of minorities in clinical trials; they also provided feedback on paper prototypes of the Health for All web tool, such as accommodation of different learning styles and the importance of engendering trust. We determined that the seven focus groups held were sufficient to capture major themes, and built the web tool on that basis. Usability testing was conducted in two iterative rounds by the think-aloud method augmented by audio recording, screen capture, and observation; we included >10 participants in each round. After resolving issues detected, we conducted beta testing with website analytics and then launched the web tool at https://healthforallproject.org/. The methods described can guide future intervention development involving adaptation of health equity tools to digital formats in community settings such as public libraries.

Prior research on clinical trials participation shows that effective methods of outreach and recruitment for underrepresented populations include direct mass mailing, personal contact and word of mouth [27]. Community involvement from partners has strong implications for retention in clinical trials among African American/Latino populations. In addition, cultural adaptation of recruitment and informational materials remains an important part of increasing participation [27]. Underlying these approaches, an establishment of trust between potential participants and the medical researchers is paramount. Through our partnerships with public library branches with deep roots in the communities they serve, our outreach methods included both personal contact and word of mouth, and branch librarians played key roles in these recruitment efforts through the strong trust placed in them by underrepresented minorities in the communities they serve. Thus, our successful recruitment efforts support these prior conclusions when engaging with underrepresented populations. Further, our results suggest that the informational setting (vs. a clinical setting) may prove as a safer space to ask questions about clinical trials and thus open the discussion forum.

Importantly, through its online platform and deployment in public libraries, Health for All is well positioned to ultimately be scaled with potential to reach the large numbers of underrepresented racial/ethnic minorities needed to impact the rates of minority enrollment in clinical research. Public libraries serve as the bedrock resource of information access and knowledge in communities across the US. Importantly, public libraries are free, widely available and often located in underserved communities where they serve as an important resource and gathering place; public libraries also provide public access to computers and the internet [11]. Thus, public libraries are valuable partners for developing educational resources aimed at improving public health, especially within medically underserved communities [11–13]. Currently, the potential reach through deployment of Health for All in seven CPL branches has been enhanced by the build out of more virtual programming by CPL, including a dedicated health and science category, and the distribution of Health for All bookmarks at CPL checkout counters. Further, the subject matter scope of Health for All has been expanded to include COVID-19, which disproportionately affects racial/ethnic minorities in the US [28].

Based on our experiences described here, we also recognize librarians as key partners in developing tools to advance health equity, such as Health for All. The librarians participating in Health for All development were seen as arbiters of factual information in their communities, and they served the role of guiding library patrons to finding answers to their questions and relevant community resources. This suggests that public librarians are well positioned to serve as trusted partners in discussing clinical trial basics with patrons. Roles played by librarians in the development of Health for All included active recruitment of library patrons to the study as well as participation in focus groups, where they shared the types of health-related questions they often are asked by patrons, their opinions of clinical trials as they relate to their patrons, and other germane information and perspectives.

During the entire design process of developing the Health for All web-based tool, it was important to always include the librarians and library patrons at the center of our approach. They collectively served as our designers by providing real-time feedback and demonstrating immediate effects of any revisions in our prototypes. Whereas other public health tools may draw from a large body of empirical databases and statistics, our process took into consideration the different factors at play in the different library environments, the dynamics between library patrons and librarian and the perceptions and experiences of the intended users.

Strengths of this study include the grounding of the Health for All web tool development (human-centered design and usability testing) in the community [9,10], the iterative [9] and contextually rich approach taken in tool development, and its predication on the Research Literacy Support flashcard tool [5]. In intervention research, favoring scientific rigor and experimental designs can come at a cost to true community-based involvement in intervention design and testing. In this paper, we report development of a web tool through human-centered design, which is inherently a community-engaged approach, in concert with examination of that process through the scientific rigor of implementation science approaches. Importantly, the process we’ve outlined, informed by implementation science, is a promising strategy that optimizes rigor and community participation and can be replicated without specialized equipment or large sample sizes.

A limitation of this study is that detailed demographic data were not captured to determine whether any participants in the human-centered design and usability testing phases were persons with disabilities or members the LGBTQ community. Another limitation regarding diversity is that there was little or no representation of Pacific Islanders or Native Americans among library patron participants, reflecting the demographic makeup of Chicago, which is predominantly Black or African American, Hispanic or Latino, and White. Acknowledging these limitations, we posit that one cannot create a tool that is genuinely optimized for all audiences. Rather, the optimization of Health for All is community based, and can serve as a starting point for subsequent platforms optimized with the inclusion of other underrepresented groups, such as the LGBTQ community or Native Americans.

## Conclusions

Inadequate diversity in clinical trials is widely recognized as a significant contributing factor to health disparities experienced by racial/ethnic minorities and other diverse populations in the US. We report here the community-based, human-centered development of Health for All, a web tool designed to improve research literacy, allay distrust of clinical research engendered by systemic racism and discrimination, and facilitate connection to clinical trials in underserved minority communities through engagement in public library settings. We anticipate that future work to investigate how a tool such as Health for All might support more virtual workflows at public libraries will be buttressed by ongoing virtual adaptations to the COVID-19 pandemic.
